# Survival of Patients with Acute Coronary Syndrome and Hematologic Malignancies—A Real-World Analysis

**DOI:** 10.3390/cancers15204966

**Published:** 2023-10-12

**Authors:** Stefan A. Lange, Christoph Schliemann, Christiane Engelbertz, Jannik Feld, Lena Makowski, Joachim Gerß, Patrik Dröge, Thomas Ruhnke, Christian Günster, Holger Reinecke, Jeanette Köppe

**Affiliations:** 1 Department of Cardiology I—Coronary and Peripheral Vascular Disease, Heart Failure, University Hospital Muenster, D-48149 Muenster, Germany; christianemaria.engelbertz@ukmuenster.de (C.E.); lena.makowski@ukmuenster.de (L.M.); holger.reinecke@ukmuenster.de (H.R.); 2 Department of Medicine A, University Hospital Muenster, D-48149 Muenster, Germany; christoph.schliemann@ukmuenster.de; 3 Institute of Biostatistics and Clinical Research, University of Muenster, D-48149 Muenster, Germany; jannik.feld@ummuenster.de (J.F.); joachim.gerss@ukmuenster.de (J.G.); jeanette.koeppe@ukmuenster.de (J.K.); 4 AOK Research Institute (WIdO), D-10178 Berlin, Germany; patrik.droege@wido.bv.aok.de (P.D.); christian.guenster@wido.bv.aok.de (C.G.)

**Keywords:** acute coronary syndrome, hematologic malignancies, comorbidities, mortality, multivariable regression analysis, real-world evidence, health services research

## Abstract

**Simple Summary:**

The impact of the co-occurrence of coronary artery disease and hematologic malignancies (HM) remains poorly understood. Therefore, the aim of this analysis was to clarify how HM affects the prognosis of acute coronary syndrome (ACS). To this end, we analyzed 439,716 patients hospitalized for ACS between 2010 and 2018, matched by age, sex, and all comorbidities for short- and long-term survival and adverse cardiac events. The incidence of ACS and HM was only 0.5%. In these patients, myelodysplastic/myeloproliferative disorders, lymphocytic leukemias, and multiple myelomas predominated. These patients were older and less likely to have an ST-segment elevation myocardial infarction. With the exception of dyslipidemia, these patients had more concomitant and previous cardiovascular disease and a worse NYHA stage. They were less likely to receive coronary angiography and percutaneous coronary intervention, although bleeding events were not significantly increased. After an adjustment for risk profile, HM was associated with lower long-term but not short-term survival.

**Abstract:**

Background: The impact of the encounter between coronary heart disease (CHD) and cancer, and in particular hematologic malignancies (HM), remains poorly understood. Objective: The aim of this analysis was to clarify how HM affects the prognosis of acute coronary syndrome (ACS). We analyzed German health insurance data from 11 regional Ortskrankenkassen (AOK) of patients hospitalized for ACS between January 2010 and December 2018, matched by age, sex and all comorbidities for short- and long-term survival and major adverse cardiac events (MACE). Results: Of 439,716 patients with ACS, 2104 (0.5%) also had an HM. Myelodysplastic/myeloproliferative disorders (27.7%), lymphocytic leukemias (24.8%), and multiple myeloma (22.4%) predominated. These patients were about 6 years older (78 vs. 72 years *). They had an ST-segment elevation myocardial infarction (STEMI, 18.2 vs. 34.9% *) less often and more often had a non-STEMI (NSTEMI, 81.8 vs. 65.1% *). With the exception of dyslipidemia, these patients had more concomitant and previous cardiovascular disease and a worse NYHA stage. They were less likely to undergo coronary angiography (65.3 vs. 71.6% *) and percutaneous coronary intervention (PCI, 44.3 vs. 52.0% *), although the number of bleeding events was not relevantly increased (*p* = 0.22). After adjustment for the patients’ risk profile, the HM was associated with reduced long-term survival. However, this was not true for short-term survival. Here, there was no difference in the STEMI patients, * *p* < 0.001. Conclusion: Survival in ACS and HM is significantly lower, possibly due to the avoidance of PCI because of a perceived increased risk of bleeding.

## 1. Introduction

Chronic inflammation associated with obesity, diabetes mellitus, hypertension, and dyslipidemia plays an important role in the development of cancer and chronic artery disease (CAD). It can be said that cancer and cardiovascular disease are two sides of the same coin [1]. Both cancer therapy and treatment options for CAD have improved significantly over the past two decades, leading to life extension [2,3]. Nevertheless, a coincidence of both disease areas leads to increased morbidity and mortality; however, patients with hematologic malignancies (HM) were not analyzed in detail in this regard [4]. In recent decades, the number of patients with HM increased worldwide, especially due to the demographic shift toward more and more elderly people [5,6,7]. Hence, this particular patient group needs our special attention.

Recent studies showed that hematologic diseases can be treated well with a variety of modern therapies [8], but when combined with myocardial infarction (MI), they lead to an excess mortality [9,10,11]. However, there are few retrospective studies in the literature that have specifically examined HM and CAD [10,11,12,13]. Thus, it should be examined how often patients with an acute coronary syndrome (ACS) are also affected by HM and what the consequences are for these patients.

For this purpose, the study at hand aimed to analyze the outcome of patients after ACS with and without concomitant HM on a large real-world cohort.

## 2. Materials and Methods

The German classification system for the reimbursement of case-related inpatient healthcare costs has its basis in the “German Diagnosis Related Groups” (G-DRG). The coding of a principal diagnosis is mandatory for all patients in hospital and is supplemented by an unlimited number of secondary diagnoses. Complications and secondary diseases are taken into account. The more complex the case, the higher the reimbursement of therapy costs.

For inpatient or outpatient cases, each diagnosis is coded according to the “German Modification of the International Statistical Classification of Diseases and Related Health Problems 10th Revision” (ICD-10 GM). In the G-DRG system, the German version of the WHO-DRG, there are more detailed coding requirements, and differentiation is made between various subgroups. For example, there are different types of hematologic disorders and different types of MI. Diagnostic, endovascular, and surgical procedures are to be coded according to the German procedure classification system (OPS).

The methods used here have been described in detail in Freisinger et al. [14] and in the supplements; all codes are found in Supplementary Table S1.

Anonymized patients data from eleven legally independent health insurance funds of the Allgemeine Ortskrankenkasse (AOK) covering around one third of German population were used. Only patients who were hospitalized for ACS (in the narrow sense ST-segment elevation myocardial infarction, STEMI and non-STEMI, NSTEMI) between 2010 and 2018 were further analyzed (Supplementary Figure S1). These patients were categorized for descriptive analysis according to the HM coded during hospitalization. Categorization was performed to obtain different patient groups as described in supplemental material.

In addition, data from the Federal Statistical Office were evaluated to determine the incidence of HM in Germany [15].

### Statistical Methods

To make an improved statement of in-hospital disease progression and therapies, a propensity score match was performed, in which a non-HM patient with ACS was assigned to each HM patient according to age, sex, and comorbidities (myocardial infarction, diabetes mellitus, dyslipidemia, peripheral artery disease, chronic kidney disease, hypertension, nicotine, chronic heart failure, and obesity).

Overall survival (OS) was estimated using a Kaplan–Meier estimator for the matched cohort. Moreover, OS was analyzed using multivariable Cox regression model including age, sex and all comorbidities, where the entire cohort was used. In contrast to the descriptive analysis, patients were not divided into different HM groups since they were part of the low sample size for several groups. Hazard ratios (HRs) and 95% confidence intervals (CIs) for all characteristics are shown in the tables and figures. All 95% CIs and all *p*-values were unadjusted standard values and descriptive only. To reflect differences between infarct entities, an interaction between HM and STEMI/NSTEMI was added in all models. Moreover, the association of guideline-recommended medication and overall survival-depending HM was analyzed using multivariable Cox regression models with time-dependent co-variables. STEMI and NSTEMI patients were analyzed separately, and an interaction between medication and HM was added.

All analyses were fully exploratory and hypothesis-generating in design and were interpreted accordingly. Data source, patient selection, statistical methods, data accessibility, and ethical approval are described in the Supplementary Materials (Text S1). This also includes applied ICD-10 GM, OPS, and ATC codes (Table S1).

In addition, it was also of interest which HM diseases are clustered in patients with ACS and to what extent this distribution pattern corresponds to the usual distribution pattern for HM in German society. Following the statistics on cancer cases in Germany regularly compiled by the Robert Koch Society, we formed the following HM groups in our analysis: Hodgkin’s disease, leukemia, multiple myeloma, and non-Hodgkin’s lymphoma.

In addition, data of the Federal Statistical Offices (Research Data Centre of the Federal Statistical Office and the Statistical Offices of the Laender, Statistisches Bundesamt [DESTATIS]; https://www.destatis.de (accessed on 23 March 2023) were analyzed to determine the incidence of HM in Germany (Supplementary Table S7).

Statistical analyses were performed using SAS software V9.4, SAS Institute Inc., Cary, NC, USA, and R version 3.6.0, R foundation, Vienna, Austria.

## 3. Results

Of 439,716 adult patients with ACS, 2104 also had a concomitant hematologic disease (0.5%). In principle, there are 478 patients per 100,000 ACS patients who also have HM. In comparison, the incidence for hematologic disorders in Germany averaged over 2011 to 2019 is 87.59 (Table S7). The distribution frequency for ACS patients with HM in descending order was as follows: Myelodysplastic and myeloproliferative disease (27.7%), lymphocytic leukemia (24.8%), multiple myeloma and malignant plasma cell neoplasms (22.4%), chronical myeloid leukemia (8.8%), aggressive lymphoma (5.9%), indolent lymphoma (5.1%), Hodgkin’s lymphoma (3.5%), and myeloid leukemia (1.9%).

### 3.1. Baseline Characteristics

In median, ACS patients with HM were six years older than patients without HM (78 vs. 72 years, see Table S2). About 35% of patients with ACS without HM had a STEMI, and 65% had a NSTEMI constellation. In contrast, the percentage of STEMI in ACS patients with HM was only 18.2%, and the vast majority of these patients had NSTEMI. ACS patients with HM had worse NYHA stage, more frequent chronic heart failure, right heart failure, and atrial fibrillation.

In addition, cardiovascular risk factors, such as diabetes mellitus (50.0% vs. 44.1%), arterial hypertension (93.9% vs. 89.5%), and psychiatric disorders (18.1% vs. 14.6%), were more prevalent in these hematologic patients. However, this was not true for obesity (27.3% vs. 29.1%), dyslipidemia (69.3% vs. 73.8%), and current nicotine abuse (14.0% vs. 21.9%). Advanced peripheral arterial occlusive disease was found more often in HM patients (7.2% vs. 2.3%). In addition, renal insufficiency (58.7% vs. 33.2%) and previous dialysis (0.9 vs. 0.7%) were more frequent in these patients.

Looking back at the medical history of these patients before ACS, patients with ACS and HM more often had a stroke (15.2% vs. 12.3%); a previously diagnosed CAD (63.8% vs. 55.5%) with, among others, a percutaneous coronary intervention (PCI) (5.8% vs. 3.6%) or bypass surgery (CABG) (10.1% vs. 7.1%); and, more often, a valve replacement (2.0% vs. 1.1%). Accordingly, these hematologic patients were more likely to have therapy with oral anticoagulants (OAC, 13.2% vs. 8.4%), platelet activation inhibitors (PAI, 26.0% vs. 20.3%), or both in combination (37.1% vs. 27.4%); statins (30.9% vs. 27.9%); beta blockers (52.7% vs. 42.1%); and angiotensin-converting enzyme inhibitors (ACE-I) or angiotensin receptor blockers (ARBs, 59.7% vs. 54.4%).

### 3.2. In-Hospital Outcome

Propensity score matching was performed for the descriptive in-hospital outcome (matched group), in which a matched partner could be assigned for 2103 patients with HM and ACS (Table S3). ACS patients with underlying HM were less likely to undergo coronary angiography (65.3% vs. 71.6%), PCI (44.3% vs. 52.0%), and DES implantation (27.5% vs. 39.5%). The ratio was reversed for BMS (12.8% vs. 9.0%). The frequency of CABG was equally distributed (6.3% vs. 5.9%). Acute renal failure (14.0% vs. 8.0%), acute renal replacement therapy (6.8 vs. 4.6%), or the combination of both (16.4% vs. 11.4%) occurred more frequently in the hematologic ACS patients. Artificial ventilation was performed approximately equally often in the two patient groups, but ventilation times were notably longer in the hematology patients (40 vs. 26 h). Bleeding events were not increased (7.9% vs. 6.9%), but blood transfusions were (40.8% vs. 13.5%). Sepsis also occurred more often in hematologic ACS patients (5.1% vs. 3.2%). In both, the length of the hospital stay was prolonged by more than 2 days on average, and the therapy costs were on average of € 1800 higher (11,285 vs. 9483 €).

### 3.3. Short-Term Mortality and Overall Survival

**Figure 1:**
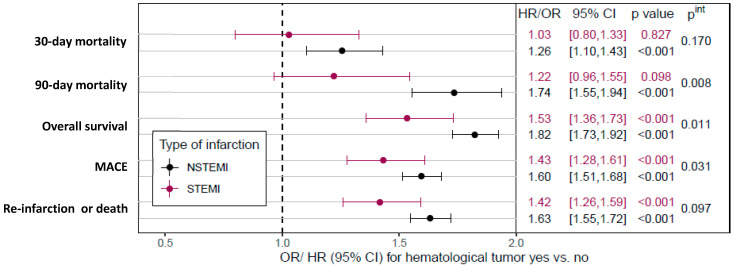
Mortality, MACE, and re-infarction/death comparison between STEMI and NSTEMI patients with and without HM. For 30- and 90-day mortality in NSTEMI, for overall survival after STEMI and NSTEMI, for MACE, and for re-infarction or death, there is a clear disadvantage in the group with HM. MACE, major adverse cardiac events; NSTEMI, non-ST-elevation myocardial infarction; STEMI, ST-elevation myocardial infarction. Abbreviations: CI, confidence interval; HR, hazard ratio; MACE, major adverse cardiovascular events; NSTEMI, non-ST-segment elevation myocardial infarction; OR, odds ratio; STEMI, ST-segment elevation myocardial infarction.

Descriptively, for the matched cohort, in-hospital mortality (16.8% vs. 14.4%), 30-day mortality (18.4% vs. 15.0%), and 90-day mortality (28.8% vs. 19.3%) were higher in ACS patients with HM (all *p* < 0.05, Supplementary Table S3). In addition, after adjustment for individual patient risk profile, HM was associated with higher 30-day and 90-day mortality in NSTEMI patients, whereas no higher risk of short-term death was observed in patients with STEMI (Figure 1).

**Figure 2:**
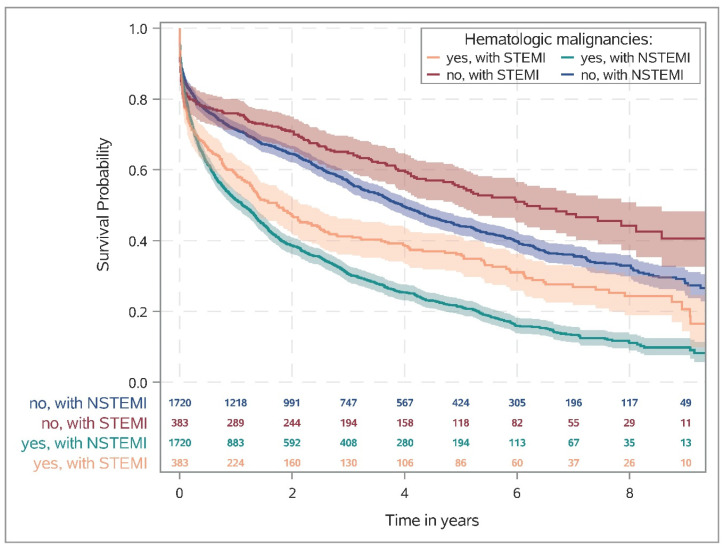
The Kaplan–Meier curves for mortality after STEMI and after NSTEMI, respectively, show that patients with STEMI have a lower probability of survival in the early phase after infarction compared with NSTEMI and a higher probability of survival in the chronic phase. Patients with hematologic malignancies, however, generally have a worse survival prognosis. Abbreviations: NSTEMI, non-ST-segment elevation myocardial infarction; STEMI, ST-segment elevation myocardial infarction.

Figure 2 shows the Kaplan–Meier survival rates for patients (matched cohort) with and without HM stratified by STEMI and NSTEMI. One year after AMI, the rate of death was almost twice as high in patients with HM as in patients without HM (STEMI: 41.3% [36.3–46.2%] vs. 24.1% [19.9–28.4%], NSTEMI: 48.5% [46.1–50.8%] vs. 28.6% [26.5–30.8%]; Supplementary Table S4). Moreover, similar effects were observed in the risk-adjusted analysis, i.e., HM was associated with lower overall survival in both STEMI and NSTEMI, with a more pronounced effect in NSTEMI (STEMI: HR 1.53, 95%CI 1.36–1.73; NSTEMI: HR 1.82, 95%CI 1.73–1.92; interaction *p*-value *p*^int^ = 0.011; Figure 1). Data on all-cause mortality, re-intervention or death, MACCE, and new cancer diagnoses are summarized in Table S4. The cumulative incidence of newly detected cancers during the follow-up period is summarized in Table S5 in the Supplementary Appendix.

**Figure 3:**
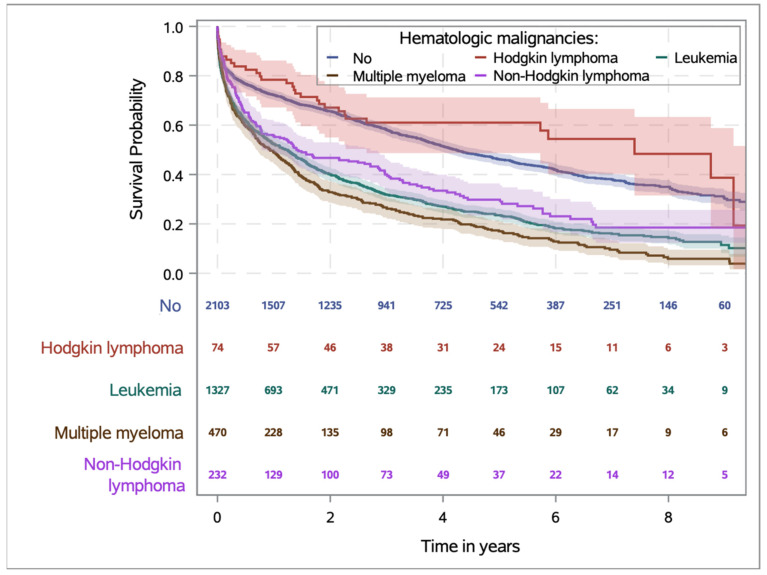
Kaplan–Meier curves for mortality after ACS in propensity-matching show that patients with ACS and hematologic malignancies have a lower probability of survival, with the exception of patients with Hodgkin’s lymphoma. Patients with multiple myeloma had the worst survival prognosis.

Overall survival stratified according to the classification of the Robert Koch Society was presented in Figure 3. Except of Hodgkin’s lymphoma, patients with ACS and HM had lower survival rates, with the poorest outcome overserved for patients with multiple myeloma. In addition, a Caplan-Meier survival diagram of ACS patients with and without hematologic malignancies is shown in the Appendix (Figure S3), based on a detailed breakdown into a total of 8 separate HM groups.

### 3.4. Guideline-Recommended Pharmaceutical Treatment during Follow-Up

**Figure 4:**
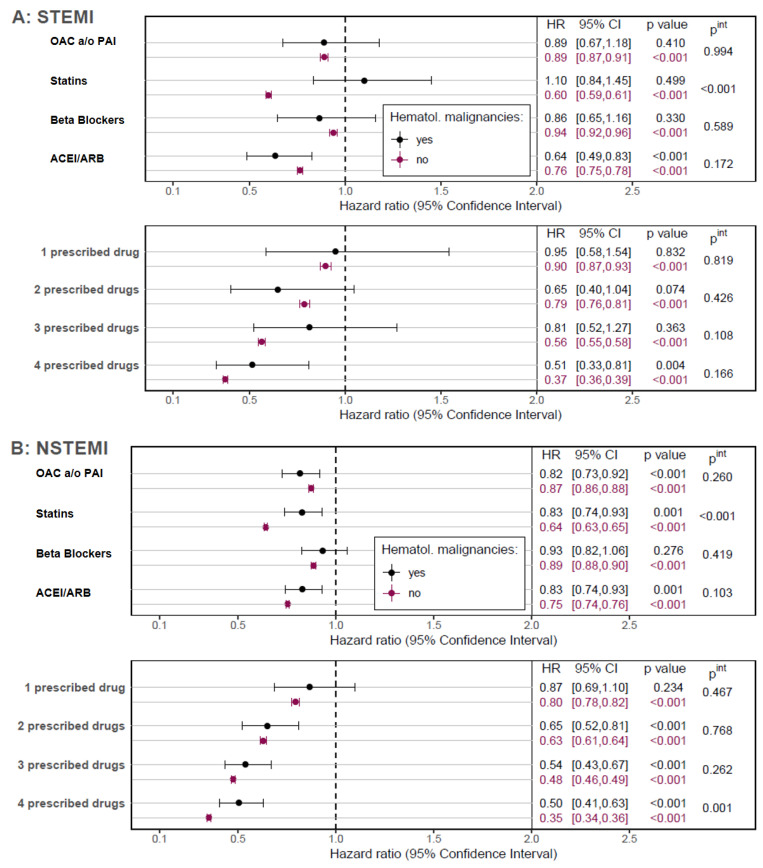
(**A**,**B**) STEMI patients who have hematologic malignancy do not benefit from statin therapy, in contrast to STEMI patients without HM. NSTEMI patients without HM benefited more from statins than NSTEMI patients with HM. ACEI, angiotensin converting enzyme inhibitor; ARB, angiotensin receptor blocker; OAC, oral anticoagulants; PAI, platelet activation inhibitor.

With the exception of oral anticoagulation and/or PAI, patients with HM after an AMI had a lower prescription rate for all medications recommended in the cardiology guidelines (Supplementary Table S6), resulting in a lower rate of patients receiving all four recommended medications (Supplementary Figure S2). Although no single effect could be demonstrated for some medications due to sample size, HM patients appeared to benefit from therapy to a similar extent as patients without HM (Figure 4). Moreover, patients without HM benefited notably more from statins in both, STEMI and NSTEMI patients (both *p*^int^ < 0.001) and the observed positive association of a four-drug therapy on OS was higher in NSTEMI-patients when compared to those with HM (*p*^int^ = 0.001).

## 4. Discussion

Within a follow-up period of up to ten years, we analyzed patient data from the AOK (Allgemeine Ortskrankenkasse), with more than 26 million insured persons in 2018 in Germany [16]. Among these, we identified a total of 439,716 patients with ACS and followed the course of their disease. Of these patients with STEMI and NSTEMI, almost 0.5% also had a malignant hematologic disease as a concomitant disease. In a comparable large retrospective analysis of U.S. residents with ACS, Mohamed et al. reported a proportion of 0.3% exclusive leukemias among all ACS patients. Other hematologic malignancies, such as the proportion of ACS patients with Hodgkin’s lymphoma, non-Hodgkin’s lymphoma, and myeloma, were not separately assessed in this study [11]. HM patients in our study were in median six years older than the other infarct patients at the time of index hospitalization. For comparison, in a large Swedish registry, patients with ACS and HM were ten years older than those without HM [10].

In addition, there are differences in the frequency of complications in patients with ACS and HM; in our study, with exception of patients with Hodgkin’s disease, all ACS patients with concomitant hematologic disorders had a worse prognosis and a disadvantage for over all-survival. This is not surprising, as these patients were more likely to experience major adverse cardiac events (MACE) or re-infarction. Although patients with HM required blood transfusions more frequently, they did not have a relevant increase in bleeding complications. This has also been described for ACS patients with leukemia in the U.S. [11]. In addition, HM patients with ACS were more likely to have acute renal failure. This is to be expected because HM patients were more likely to have chronic renal failure in our study as well as in the data analysis from the U.S. [11]. Consequently, it is not unexpected that ACS patients with HM had higher risk for thirty- and ninety-day mortality when diagnosed with NSTEMI. Surprisingly, however, these mortality differences were not detectable in STEMI.

Comparing the survival probabilities of patients with hematologic malignancies in general provided by the Robert Koch Institute and also U.S. Cancer Statistics and comparing them with our study, the following results are obtained with regard to the relative 5-year survival rates [17,18]: For Hodgkin’s disease, relative survival rates range from 81–91%. In contrast, patients in our study with Hodgkin’s lymphoma and ACS had a 5-year survival rate of 60%. In non-Hodgkin’s lymphoma, 5-year survival rates generally range from 70–72%. In our analyses, patients with non-Hodgkin’s lymphoma and ACS had a 5-year survival rate of only 30%. In multiple myeloma, 5-year relative survival rates are particularly poor, ranging from 54–56% and less than 20% in the case of ACS. For leukemia (without further differentiation), 5-year survival rates in Germany are 56–58% [17]. In the U.S., 5-year survival rates, depending on the type of leukemia, range from 24% in AML to 86% in CLL [18]. In our analysis, patients with leukemia and ACS had a 5-year survival rate reduced by half (to about 25%). In summary, the patients presented here who have a hematological disease in addition to myocardial infarction had significantly worse overall survival.

It must be emphasized that patients with a diagnosis of NSTEMI had a lower probability of survival compared with STEMI in the chronic course, both in patients with and without a diagnosis of HM. Five years after NSTEMI, the probability of survival is about 20% in patients with HM, whereas it was approximately 45% without HM. The results for ACS patients in general contradict French data that showed a disadvantage for STEMI patients compared with NSTEMI patients in the first 28 days and saw no difference for 10-year survival. However, only patients from 2006 were included here [19]. A study from South Korea on patients with and without diabetes and STEMI or NSTEMI came to similar results as ours, albeit only at a 24-month follow-up [20]. Possible reasons for the poorer outcome for NSTEMI patients with HM in our study are the greater number of relevant comorbidities (e.g., atrial fibrillation, diabetes, and hypertension), the lower proportion of invasive coronary diagnostics and therapies, and, last but not least, the higher average age at the time of infarction.

Another possible explanation for the difference in mortality could also be the lower implementation of ACS guidelines. For example, in our study, HM patients with ACS were less likely to be prescribed all four guideline-compliant ACS medications, particularly within the first two years after ACS. There also appear to be differences in the effect of ACS medication. Thus, to our general surprise, the benefit of statin therapy was reduced in patients with NSTEMI and HM and even completely abolished in STEMI patients with HM. This observation is diametrically opposed to the survival benefit that statin therapy usually entails in ACS patients [21]. This is also in contrast to the expected statin effect of inhibiting tumor proliferation and metastasis in various cancers by lowering cholesterol [22,23,24,25,26]. For multiple myeloma, which was more common in ACS patients in our study, both a statin-sensitive and a statin-insensitive myeloma cell line have been described in the past, in which statin therapy had an apoptotic effect [27]. An argument that could support the observations made in our study is that cholesterol is also crucial for the synthesis of biological membranes and is a component of plasma lipoproteins, which serve as energy stores in animals [28]. It is possible that in the constellation of transmural myocardial infarction and HM, the function of cholesterol as an energy store and source becomes more prominent.

In the current ACS guideline of the European Society of Cardiology, the routine administration of beta-blockers to all ACS patients regardless of LVEF is still a class IIa indication and should continue to be considered [29]. In our study beta-blocker therapy had no effect on the survival of STEMI and NSTEMI patients with hematological malignancies. This makes the observation that the survival probability of STEMI patients with hematologic malignancies can only be achieved with a four-drug combination of post-myocardial infarction drugs all the more surprising. A possible explanation for this could also be the lower proportion of STEMI among HM patients in absolute terms. However, even if a really large database were analyzed, only 383 patients with STEMI and HM could be included, which made the estimation of the treatment effects more difficult.

Among the patients with ACS and hematologic disorders presented here, patients with leukemia are the most common, accounting for 63.2%. Among the leukemia subtypes, in turn, myelodysplastic syndrome and myeloproliferative syndrome ranked first. This distribution differs from the data on leukemia in Germany collected by the Robert Koch Institute. Instead, the Robert Koch Institute showed the following distribution pattern for leukemia: Chronic lymphatic leukemia (CLL) as the most common form, followed by acute myeloid leukemia (AML), other, chronic myeloid leukemia (CML), and acute lymphocytic leukemia (ALL) [17].

Multiple myeloma (including malignant plasma cell neoplasms) was represented in our study with 22.4%, followed by non-Hodgkin’s lymphoma with 11.0% and Hodgkin’s lymphoma with 3.5%. A Swedish analysis among cancer patients demonstrated a clustered occurrence of acute coronary syndromes in patients with multiple myeloma during the first 10 years after diagnosis [13].

It can be concluded that patients with ACS have a different frequency distribution with respect to concomitant hematologic diseases than the general population. The older age of this patient group may also underlie this observation. A shift in the frequencies of hematologic cancers according to population age can also be seen in the regular publications of U.S. Cancer Statistics [18]. However, as in the RKI statistics, this statistic also showed a clustering of non-Hodgkin’s lymphomas in the group over 65 years of age. Hypothetically, it could also be a matter of similarities in the development of both diseases, CHD and myeloproliferative disorders.

## 5. Limitations

The generally known limitations of a retrospective study design apply to our data analysis, which include the risk of selection and information bias [30]. A disadvantage of our study is also the lack of laboratory values of the patients included here. Thus, we had to rely on the coded diagnoses and cannot verify them individually. Nevertheless, a high data quality can be assumed here because our study focused on hard endpoints, such as acute myocardial infarction and mortality, which are very unlikely to have been coded incorrectly because they have a direct and relevant impact on billing. Precise rules for the coding of principal and secondary diagnoses and procedures were used, which have remained unchanged for more than 20 years in Germany and also with respect to the diagnoses analyzed in this study. Possible incomplete coding is very unlikely, as complete coding is required for a complete reimbursement of treatment costs. We also have no information on individual drug dosage. Thus, we must assume that the dosage was in accordance with the medication insert and the guideline, but we cannot verify this.

In addition, legal requirements and the integrated control system guarantee a very high reliability of the coded database. However, our analysis is purely observational and does not allow for any conclusion about causal interactions.

Eleven independent regional health insurance funds, which are distributed throughout Germany and thus cover healthcare, form the AOK. Membership in the AOK is open to every resident, regardless of profession, income, age, or state of health. Nevertheless, there are likely to be differences between regions and states, which may also result in different healthcare coverage depending on regional health insurance. This may influence the data, but it shows area-wide healthcare coverage in a real cohort. Unlike randomized trials, observational studies, or registries, health service data are not subject to selection by the commissioner or the implementer.

## 6. Conclusions

Cancer and cardiovascular diseases are among the most severe and life-limiting diseases in the economically prosperous countries of the world. As already shown, a coincidence of both diseases significantly reduces the survival of patients markedly. For the occurrence of ACS in association with hematologic malignant disease, little data are available that have adequately investigated this issue. The present retrospective study aimed to shed light on this issue and, therefore, focused on various forms of malignant hematologic disorders that occurred in patients with an ACS as a co-diagnosis. Thus, the mortality of ACS patients increased notably by HM with the exception of Hodgkin’s disease regardless of older age. In addition, a different distribution of the various leukemia types was found for the ACS patients compared to the normal population. However, not only did subtypes of leukemia occur differently, but also multiple myeloma was overrepresented in ACS patients. Not only were patients with ACS and HM less likely to have obesity and dyslipidemia, but also the effect of statin-lowering therapy was attenuated or, as in STEMI patients with HM, absent. Understandably, many questions remain unanswered, and we still owe explanations. Our study also failed to show an accumulation of adverse bleeding events for HM patients, which justifies the progressive invasive management of infarction in these patients as well. In conclusion, our analysis showed worse long-term survival for patients with NSTEMI in general and also in association with HM compared with STEMI.

## Data Availability

The authors confirm that the data utilized in this study cannot be made available in the manuscript, the supplemental files, or in a public repository due to German data protection laws (‘Bundesdatenschutzgesetz’, BDSG). Therefore, they are stored on a secure drive in the AOK Research Institute (WIdO) to facilitate replication of the results. Generally, access to data of statutory health insurance funds for research purposes is possible only under the conditions defined in German Social Law (SGB V § 287). Requests for data access can be sent as a formal proposal specifying the recipient and purpose of the data transfer to the appropriate data protection agency. Access to the data used in this study can only be provided to external parties under the conditions of the cooperative contract of this research project and after written approval by the sickness fund. For assistance in obtaining access to the data, please contact wido@wido.bv.aok.de.

## Supplementary Materials

The following supporting information can be downloaded at: https://www.mdpi.com/article/10.3390/cancers15204966/s1, Text S1: Material and Methods (comprehensive information); Figure S1: CONSORT flow-chart; Figure S2: Prescription frequency of the four cardiovascular drugs * during the follow-up period in patients with STEMI or NSTEMI w/wo hematological malignancies; Figure S3: Caplan-Meier Survival in ACS depending on hematology malignancies; Figure S4: Survival in STEMI vs. NSTEMI; Table S1: Codes for data retrieval; Table S2: Baseline characteristics, stratified by presence or absence of hematology malignancies; Table S3: In-hospital outcome depending on the presence or absence of hematological malignancies; Table S4: Eventraten–matched cohort depending on the presence or absence of hematological malignancies distinguished between STEMI and NSTEMI; Table S5: Newly detected cancers during follow-up; Table S6: Drug adherence during the follow-up period based on prescribed medications. Table S7. Incidence for various hematologic malignancies divided into 8 subgroups in Germany from 2011 to 2019.
