# Supplementary material for: Survival of Patients with Acute Coronary Syndrome and Hematologic Malignancies—A Real-World Analysis
Source: Cancers (Basel). 2023 Oct 12;15(20):4966. doi: 10.3390/cancers15204966 (PMC10605274; doi:10.3390/cancers15204966)
Supplement: Supplementary file 1 [file cancers-15-04966-s001.zip › cancers-2632430-supplementary.pdf]

Text S1. Material and Methods (comprehensive information)

The German remuneration system is based on the system of the "German Diagnosis Related Groups" (G-DRG). This requires the coding of a main diagnosis for all inpatients, which must be carefully selected after discharge, taking into account the underlying reason for hospital admission. In addition, an unlimited number of secondary diagnoses can be coded to reflect co-morbidities and complications, whether present or during the hospital stay. These secondary diagnoses increase the patient's comorbidity and complexity and have some influence on reimbursement. Each diagnosis has to be coded according to the "German Modification of the International Statistical Classification of Diseases and Related Health Problems 10th Revision" (ICD-10 GM). In addition to the WHO ICD-10, some diagnoses are more detailed in the German Version due to the coding requirements of the G-DRG-System. This enables the separation of subgroups such as various types of cancer, as well as acute ST-segment elevation myocardial infarction (STEMI) and non-STEMI. Similar to the ICD for diagnosis, all diagnostic, endovascular and surgical procedures must be coded according to the German procedure classification ("Operationen und Prozedurenschlüssel", OPS). Most of them have direct impact on reimbursement.

Using the coded diagnoses and procedures, each case is then assigned to a specific G-DRG, depending on its main diagnosis and combination of secondary diagnoses and procedures, and induces a certain reimbursement of costs.

All applied ICD-10 GM and OPS codes are listed in **Table S1.**

Due to the high influence of diagnoses and procedures on reimbursement, around 30% of all cases are checked and corrected by independent medical working groups ("Medizinischer Dienst").

Data Source

The Allgemeine Ortskrankenkasse (AOK) is an amalgamation of 16 regional health insurances, which together form the largest statutory health insurance in Germany. The AOK is currently responsible for more than 26 million people, around 30% of the entire German population. All patient data is stored in a central IT database of the AOK Research Institute (WIdO, Berlin), from which we receive aggregated and anonymized data of all patients who meet the following criteria.

Patient Selection

All patients with an age ≥18 years were hospitalized with a coded main diagnosis STEMI (ICD-10 GM code I21.0, I21.1; I21.2, I22.0, I22.1, I22.8) in 2010 up to 2018 were included in the analysis (**Figure S1**). This hospitalization was defined as the index hospitalization. Patients were divided into different hematology disease subgroups (group 1–8) according to a HD diagnosis during the Index hospital stay or in the previous 2 years, as follows: Hodgkin-Lymphoma [Lymphogranulomatosis] {group 1}, C81.-; Follicular lymphoma, Non-Follicular lymphoma {group 2}, C82.-; C83.0; Aggressive lymphoma {group 3}, C83.3; C83.5; C83.7; C83.8; Plasmocytoma and malignant plasma cell neoplasm {group 4}, C90.x0; C90.x1; Lymphocytic leukemia {group 5}, C91.10; C91.11; Myeloid leukemia, monocytic leukemia, other leukemias of specified cell type {Group 6}, C92.3x; C92.5x; C92.6x; C92.7x; C92.8x; C92.9x; C93.0x; C93.7x; C94.0x; C94.2x; Chronic myeloid leukemia {Group 7}, C92.1x; and Myelodysplastic and myeloproliferative diseases, unclassifiable, Myelodysplastic syndromes {Group 8}. and no Cancer (no C* code in history); **Figure S2**.

The baseline characteristics also included other coded diagnoses, such as hypertension, diabetes mellitus, dyslipidemia, atrial fibrillation, metastasis at or within two years of index hospitalization, and procedures such as percutaneous coronary intervention within two years of index hospitalization.

In-Hospital Treatment, Outcome, Medication and Follow-up

All coded interventions during the hospital stay as well as the diagnoses shock, death, stroke, bleeding, sepsis and acute kidney failure were considered as inpatient treatment or outcome.

Patients were followed up for up to nine years after index hospitalization.

Statistical Methods

Patient data were grouped by the cancer type that was coded for descriptive analysis during their baseline phase. In order to obtain different patient groups, the grouping was carried out in a hierarchical manner, which is shown in the **Figure S2**. Patients with Lymphocytic leukemia were placed in the Lymphocytic leukemia group if they had Lymphocytic leukemia and no Myeloid leukemia. Patients were placed in the lung cancer group if they had lung cancer and no prostate or breast cancer, etc. The exact hierarchical order was prostate cancer> breast cancer> lung cancer> colon cancer> urinary tract cancer> skin cancer> no cancer. Qualitative data were tested with a two-tailed chi-square test and quantitative data with a two-tailed Wilcoxon test. The 8-year overall survival rate (OS) was estimated using a Kaplan-Meier estimator; all p-values of the test procedures described above are purely descriptive and not adjusted.

The endpoint OS was analyzed using the multivariable Cox regression model. The models included baseline patient risk profiles. In contrast to the descriptive analysis, the patients were not divided into different cancer groups and each patient can have several types of cancer in his risk profile. All presented 95% confidence intervals (CIs) and p-values are by default unadjusted and purely descriptive. The hazard ratios (HRs) and the unadjusted 95%CI for all characteristics are shown in the tables and figures. All analyzes should be fully exploratory (generating hypotheses), non-confirmatory and will be interpreted accordingly. Statistical analyzes were carried out with R version 3.6.0 (2019-04-26), R foundation, Vienna, Austria.

Data Accessibility

The authors confirm that the data utilized in this study cannot be made available in the manuscript, the supplemental files, or in a public repository due to German data protection laws ("Bundesdatenschutzgesetz", BDSG). Therefore, they are stored on a secure drive in the AOK Research Institute (WIdO), to facilitate replication of the results. Generally, access to data of statutory health insurance funds for research purposes is possible only under the conditions defined in German Social Law (SGB V § 287). Requests for data access can be sent as a formal proposal specifying the recipient and purpose of the data transfer to the appropriate data protection agency. Access to the data used in this study can only be provided to external parties under the conditions of the cooperation contract of this research project and after written approval by the sickness fund. For assistance in obtaining access to the data, please contact wido@wido.bv.aok.de.

Ethical Vote

The data available here were evaluated in the GenderVasc research project. This project was approved by the ethics committee of the Landesaerztekammer Westfalen-Lippe and the medical faculty of the Westphalian Wilhelms University of Muenster (No 2019-21-f-S).

Table S1. Codes for data retrieval.

International Classification of Disease 10th Revision, German Modification (ICD-10 GM)	Code
Acute STEMI	I21.0, I21.1; I21.2, I22.0, I22.1, I22.8
NSTEMI	I21.4, I21.9
Previous myocardial infarction (MI)	I21.-; I22.-
Cerebrovascular Disease (CVD)	I65.-; I66.-; I67.2
Previous stroke	I63.-; I64.-; I69.3; I69.4
Hypertension	I10.-; I11.-; I12.-; I13.-; 15.-

Diabetes mellitus (DM)	E10.-; E11.-; E12.-; E13.-; E14.-
Dyslipidemia	E78.-
Obesity	E66.-
Smoking	F17.-
Artrial fibrillation (AF) and flutter (AFL)	I48.-
PAD 1–3	I70.20; I70.21; I70.22 (from 2015)
PAD 4–6	I70.22 (until 2015);<br>I70.23; I70.24; I70.25 (from 2015)
Chronic heart failure (CHF)	I50.-
Chronic kidney disease (CKD)	N18.-; N19.-
Cancer	C00.- to C97.-
Colon Cancer	C18.-; C19.-; C20.-; C21.-
Lung Cancer	C34.-
Skin Cancer	C43.-; C44.-
Breast Cancer	C50.-
Prostate Cancer	C61.-
Urinary Tract Cancer	C64.-; C65.-; C66.-; C67.-; C68.-
Hodgkin-Lymphoma [Lymphogranulomatosis] {group 1}	C81.-
Follicular lymphoma, Non-Follicular lymphoma {group 2}	C82.-; C83.0
Aggressive lymphoma {group 3}	C83.3; C83.5; C83.7; C83.8
Plasmocytoma and malignant plasma cell neoplasm {group 4}	C90.x0; C90.x1
Lymphocytic leukemia {group 5}	C91.10; C91.11
Myeloid leukemia, monocytic leukemia, other leukemias of specified cell type {Group 6}	C92.3x; C92.5x; C92.6x; C92.7x; C92.8x; C92.9x; C93.0x; C93.7x; C94.0x; C94.2x
Chronic myeloid leukemia {Group 7}	C92.1x
Myelodysplastic and myeloproliferative diseases, unclassifiable, Myelodysplastic syndromes {Group 8}	C94.6x; D46.-
Diseased Coronary Vessels: 1	I25.11 (and no I25.12 or I25.13)
Diseased Coronary Vessels: 2	I25.12 (and no I25.13)
Diseased Coronary Vessels: 3	I25.13
Shock	T81.1; R57.0
Acute stroke	I63.-; I64.-
Hemorhagic stroke	I60.-; I61.-; I62.-
Bleeding	K92.-; H44.8; T81.0; T81.2; T81.3; T81.7
Sepsis	A41;
Acute Renal Failure (ARF)	N17;
Previous CABG	Z95.1 (with additional OPS codes)
Previous heart Valve implantation	Z95.2 (with additional OPS codes)
Metastasis	C77.-; C78.-; C79.-
**German procedure classification system (OPS)**	
Blood transfusion	8-800.0; 8-800.1; 8-800.c
PCI (previous or current)	8-837;
Current CABG	5-36 (with additional ICD codes)
Current valve implantation	5-351; 5-352; 5-353; 5-354 (with additional ICD codes)

Abbreviations: AF, atrial fibrillation; AFL, atrial flutter; ARF, Acute renal failure; CABG, coronary artery bypass grafting; CHF, chronic heart failure; CKD, chronic kidney disease; CVD, cerebrovascular disease; DM, diabetes mellitus; MI, myocardial infarction; OPS, Operationen und Prozedurenschluessel; PAD, peripheral artery disease; PCI, percutaneous coronary intervention; STEMI, ST-elevation myocardial infarction.

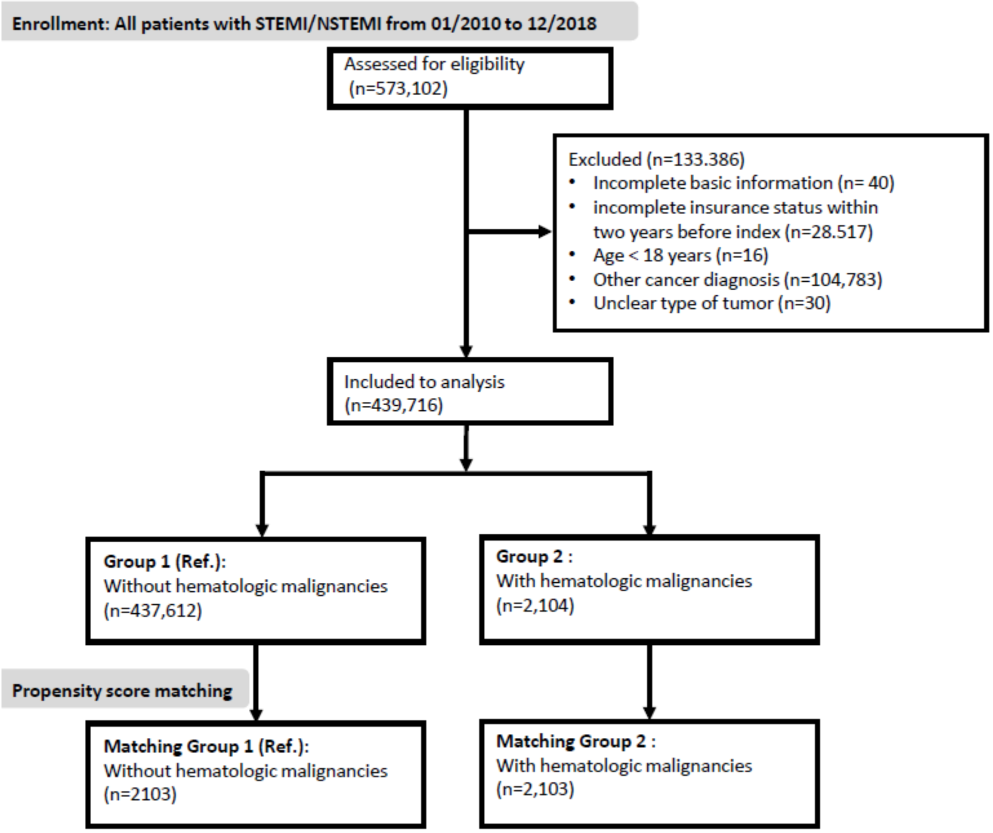

Figure S1. CONSORT flow-chart. Cohort: all adult patients, with STEMI/NSTEMI as HD in 2010 - 2018. Classification hematological tumors (yes/no) and group classification (8 groups). Tumor group determined from secondary diagnoses in case, if group not clear, then exclusion (see Figure 1). Also all with other cancer diagnosis excluded (control group is completely without C-diagnosis).

Table S2. Baseline characteristics, stratified by presence or absence of hematology malignancies.

	**Hematologic Malignancies**		***p* value**
	**No (Control Group)**	**Yes**	
**Frequency—*n* (%)**	**437,612 (99.5%)**	**2104 (0.5%)**	**n.a.**
Type of tumor—*n* (%)			
Group 1	0 (0.0%)	74 (3.5%)	
Group 2	0 (0.0%)	108 (5.1%)	
Group 3	0 (0.0%)	124 (5.9%)	
Group 4	0 (0.0%)	471 (22.4%)	n.a.
Group 5	0 (0.0%)	521 (24.8%)	
Group 6	0 (0.0%)	39 (1.9%)	
Group 7	0 (0.0%)	185 (8.8%)	
Group 8	0 (0.0%)	582 (27.7%)	
Female sex—*n* (%)	172,638 (39.5%)	809 (38.5%)	0.349
Median age (Q1, Q3)	72 (60, 81)	78 (72, 83)	<0.001
STEMI—*n* (%)	152,853 (34.9%)	383 (18.2%)	<0.001
NSTEMI—*n* (%)	284,759 (65.1%)	1721 (81.8%)	
Psychiatric diseases—*n* (%)	63,865 (14.6%)	381 (18.1%)	<0.001
Chronic kidney disease—*n* (%)	145,187 (33.2%)	1235 (58.7%)	<0.001
Previous Dialysis—*n* (%)	2889 (0.7%)	18 (0.9%)	<0.001
No. of diseased coronary vessels—*n* (%)			
unknown	96,825 (22.1%)	707 (33.6%)	
1	89,691 (20.5%)	286 (13.6%)	<0.001
2	93,504 (21.4%)	343 (16.3%)	
3	157,592 (36.0%)	768 (36.5%)	

Peripheral artery disease—*n* (%)			
No LEAD	383,678 (87.7%)	1743 (82.8%)	<0.001
LEAD RF stage 1–3	34,825 (8.0%)	221 (10.5%)	
LEAD RF stage 4–6	19,109 (4.4%)	140 (6.7%)	
Left ventricular heart failure—*n* (%)			
No LV-CHF	244,563 (55.9%)	840 (39.9%)	<0.001
NYHA I	11,317 (2.6%)	38 (1.8%)	
NYHA II	36,267 (8.3%)	186 (8.8%)	
NYHA III	58,587 (13.4%)	357 (17.0%)	
NYHA IV	86,878 (19.9%)	683 (32.5%)	
Right ventricular heart failure—*n* (%)	59,883 (13.7%)	566 (26.9%)	<0.001
Chronic heart failure—*n* (%)	230,135 (52.6%)	1477 (70.2%)	<0.001
Previous cerebrovascular disease—*n* (%)	54,254 (12.4%)	377 (17.9%)	<0.001
Atrial fibrillation—*n* (%)	112,796 (25.8%)	820 (39.0%)	<0.001
Diabetes mellitus—*n* (%)	193,080 (44.1%)	1051 (50.0%)	<0.001
Dyslipidemia—*n* (%)	323,101 (73.8%)	1457 (69.3%)	<0.001
Obesity—*n* (%)	127,467 (29.1%)	575 (27.3%)	0.070
Current smoking—*n* (%)	95,723 (21.9%)	295 (14.0%)	<0.001
Hypertension—*n* (%)	391,858 (89.5%)	1976 (93.9%)	<0.001
Previous CAD—*n* (%)	242,794 (55.5%)	1343 (63.8%)	<0.001
Previous stroke—*n* (%)	53,796 (12.3%)	320 (15.2%)	<0.001
Previous AMI—*n* (%)	40,471 (9.3%)	237 (11.3%)	0.002
Previous PCI—*n* (%)	15,922 (3.6%)	122 (5.8%)	<0.001
Previous CABG—*n* (%)	31,040 (7.1%)	212 (10.1%)	<0.001
Previous valve replacement—*n* (%)	4829 (1.1%)	43 (2.0%)	<0.001
**Previous medication:**			
Previous OAC—*n* (%)	36,820 (8.4%)	278 (13.2%)	<0.001
Previous PAI—*n* (%)	88,953 (20.3%)	546 (26.0%)	<0.001
Previous OAC or/and PAI—*n* (%)	119,965 (27.4%)	780 (37.1%)	<0.001
Previous statins—*n* (%)	122,030 (27.9%)	649 (30.9%)	0.003
Previous beta blockers—*n* (%)	184,040 (42.1%)	1109 (52.7%)	<0.001
ACE- inhibitors/ ARB—*n* (%)	238,002 (54.4%)	1257 (59.7%)	<0.001

For better comparability, we did all univariate analyses with a matched cohort: the following factors are included: Sex, Group_myocardial infarction, age_index, year_index, DM, Dyslipidemia, PAD, CKD, Hypertension, Nicotin, CHF, and Obesity. Abbreviations: Angiotensin-converting enzyme, ACE; acute myocardial infarction, AMI; angiotensin receptor blocker, ARB; coronary artery bypass grafting, CABG; coronary artery disease, CAD; chronic heart failure, CHF; *lower extremity arterial disease*, LEAD; numbers, *n*; non-ST elevation myocardial infarction, NSTEMI; New York heart association, NYHA; oral anticoagulants, OAC; platelet activation inhibition, PAI; percutaneous coronary intervention, PCI; Rutherford, RF; ST elevation myocardial infarction, STEMI.

Table S3. In-hospital outcome depending on the presence or absence of hematological malignancies.

	Hematologic Malignancies			
	**No (Control Group)**	**No (Matched Group)**	**Yes (Matched Group)**	***p* value**
	437,612 (99.5%)	2103 (50.0%)	2103 (50.0%)	**n.a.**
Diagnostic coro	350,440 (80.1%)	1506 (71.6%)	1374 (65.3%)	<0.001
PCI—*n* (%)	277,253 (63.4%)	1094 (52.0%)	931 (44.3%)	<0.001
DES—*n* (%)	207,237 (47.4%)	831 (39.5%)	579 (27.5%)	<0.001
Only BMS—*n* (%)	51,943 (11.9%)	190 (9.0%)	270 (12.8%)	<0.001
CABG—*n* (%)	28,331 (6.5%)	124 (5.9%)	133 (6.3%)	0.562
Any intervention—*n* (%)	354,488 (81.0%)	1516 (72.1%)	1389 (66.0%)	<0.001
IABP	7712 (1.8%)	35 (1.7%)	25 (1.2%)	0.194
ECMO	3463 (0.8%)	14 (0.7%)	13 (0.6%)	0.847
Acute renal failure	30,212 (6.9%)	186 (8.8%)	294 (14.0%)	<0.001
Renal replacement therapy	13,761 (3.1%)	97 (4.6%)	142 (6.8%)	0.003
renal failure a./o. replacement therapy	35,751 (8.2%)	239 (11.4%)	346 (16.4%)	<0.001

Shock	34,467 (7.9%)	168 (8.0%)	160 (7.6%)	0.646
Schock_res_LV_sup	39,105 (8.9%)	189 (9.0%)	174 (8.3%)	0.410
GpIIb_IIIa_In	53,518 (12.2%)	169 (8.0%)	117 (5.6%)	0.001
ischemic stroke	7229 (1.7%)	42 (2.0%)	43 (2.0%)	0.913
Ventilation:				
n (%)	65,699 (15.0%)	363 (17.3%)	393 (18.7%)	0.228
Median time—h (Q1,Q3)	35 (7, 146)	26 (6, 126)	40 (8, 157)	0.032
resuscitation	30,280 (6.9%)	142 (6.8%)	124 (5.9%)	0.254
Blood transfusion	44,656 (10.2%)	283 (13.5%)	858 (40.8%)	<0.001
Bleeding event	29,121 (6.7%)	145 (6.9%)	166 (7.9%)	0.216
Bleeding or blood transfusion	63,234 (14.5%)	372 (17.7%)	901 (42.8%)	<0.001
Sepsis	9505 (2.2%)	67 (3.2%)	108 (5.1%)	0.002
Mean length of stay—days (±SD)	12.2 (± 14.2)	13.9 (± 14.9)	17.5 (± 18.0)	<0.001
Mean charges—EUR (±SD)	8994.14 (± 15301.74)	9482.66 (± 14860.99)	11285.45 (± 19343.52)	<0.001
Inhospital death	52,660 (12.0%)	302 (14.4%)	353 (16.8%)	<0.001
30-day mortality	55,596 (12.7%)	314 (15.0%)	386 (18.4%)	0.003
90-day mortality	68,619 (15.7%)	405 (19.3%)	604 (28.8%)	<0.001

Abbreviations: Bare metal stent, BMS; coronary artery bypass grafting, CABG; coronary angiography, CORO; drug eluting stent, DES; extra-corporal membrane oxygenation, ECMO; glycoprotein, GP; intra-aortic balloon pump, IABP; percutaneous coronary intervention, PCI; quartile, Q; standard deviation, SD.

Table S4. Event rates—matched cohort depending on the presence or absence of hematological malignancies distinguished between STEMI and NSTEMI.

	**STEMI—Hematologic Malignancies**		**NSTEMI—Hematologic Malignancies**	
	**No**	**Yes**	**No**	**Yes**
**Overall Death—% (95% CI)**				
1 year rate	24.1% (19.9–28.4%)	41.3% (36.3–46.2%)	28.6% (26.5–30.8%)	48.5% (46.1–50.8%)
2 years rate	29.2% (24.7–33.9%)	53.1% (47.9–58.0%)	35.5% (33.2–37.8%)	61.4% (59.0–63.7%)
3 years rate	35.3% (30.4–40.2%)	58.8% (53.5–63.6%)	43.4% (41.0–45.8%)	69.4% (67.1–71.6%)
4 years rate	40.3% (35.1–45.4%)	61.5% (56.3–66.4%)	50.4% (47.8–52.9%)	74.6% (72.3–76.8%)
5 years rate	44.8% (39.3–50.2%)	63.8% (58.5–68.7%)	55.9% (53.2–58.5%)	78.7% (76.4–80.8%)
6 years rate	49.0% (43.1–54.6%)	69.0% (63.4–73.8%)	60.2% (57.5–62.9%)	84.1% (81.8–86.1%)
7 years rate	52.6% (46.2–58.5%)	73.0% (67.2–78.0%)	64.1% (61.2–66.8%)	86.7% (84.3–88.7%)
8 years rate	55.8% (48.7–62.4%)	75.7% (69.5–80.8%)	67.0% (63.9–69.9%)	88.9% (86.4–91.0%)
9 years rate	59.4% (51.0–66.9%)	79.3% (71.4–85.3%)	72.0% (68.2–75.4%)	90.2% (87.4–92.3%)
**Reinfarction or death—% (95% CI)**				
1 year rate	32.4% (27.8–37.2%)	48.8% (43.7–53.7%)	37.9% (35.6–40.2%)	57.4% (55.0–59.7%)
2 years rate	38.2% (33.3–43.1%)	59.9% (54.7–64.7%)	45.8% (43.4–48.1%)	69.0% (66.8–71.2%)
3 years rate	42.2% (37.1–47.2%)	65.4% (60.2–70.0%)	53.6% (51.1–56.0%)	75.8% (73.6–77.8%)
4 years rate	47.5% (42.1–52.6%)	68.6% (63.4–73.2%)	60.1% (57.6–62.6%)	79.9% (77.8–81.9%)
5 years rate	52.9% (47.3–58.2%)	71.8% (66.6–76.4%)	65.2% (62.6–67.6%)	83.6% (81.5–85.4%)
6 years rate	58.4% (52.3–64.0%)	76.6% (71.3–81.1%)	69.2% (66.6–71.7%)	88.0% (85.9–89.8%)
7 years rate	62.0% (55.5–67.8%)	78.9% (73.5–83.4%)	72.2% (69.4–74.7%)	90.0% (87.9–91.8%)
8 years rate	64.9% (57.9–70.9%)	81.8% (75.8–86.4%)	75.2% (72.3–77.9%)	91.3% (89.1–93.0%)
9 years rate	70.7% (59.7–79.3%)	83.8% (76.5–89.0%)	78.9% (75.4–81.9%)	92.6% (90.1–94.4%)
**MACCE—% (95% CI)**				
1 year rate	34.8% (30.0–39.6%)	50.9% (45.8–55.8%)	40.0% (37.7–42.3%)	58.9% (56.6–61.2%)
2 years rate	40.6% (35.6–45.5%)	61.5% (56.3–66.2%)	48.4% (46.0–50.7%)	70.4% (68.2–72.6%)
3 years rate	43.9% (38.8–48.8%)	66.7% (61.6–71.3%)	56.3% (53.9–58.7%)	77.3% (75.1–79.2%)
4 years rate	50.2% (44.8–55.3%)	71.0% (65.9–75.5%)	62.4% (59.9–64.8%)	81.2% (79.1–83.1%)
5 years rate	56.0% (50.4–61.3%)	74.2% (69.1–78.6%)	67.3% (64.7–69.7%)	84.7% (82.7–86.5%)
6 years rate	60.9% (54.9–66.3%)	78.9% (73.7–83.2%)	71.6% (69.0–74.1%)	89.5% (87.5–91.2%)
7 years rate	63.6% (57.3–69.2%)	81.8% (76.5–86.0%)	74.5% (71.8–77.0%)	91.8% (89.8–93.4%)
8 years rate	66.4% (59.7–72.3%)	84.0% (78.1–88.4%)	76.9% (74.1–79.5%)	93.2% (91.1–94.8%)
9 years rate	72.0% (61.4–80.2%)	88.2% (78.6–93.6%)	80.7% (77.2–83.7%)	94.2% (91.8–95.9%)

**New cancer diagnosis—% (95% CI)**				
1 year rate	1.3% (0.5–2.9%)	13.3% (10.1–16.9%)	1.5% (1.0–2.1%)	15.2% (13.6–17.0%)
2 years rate	2.4% (1.2–4.3%)	16.9% (13.3–20.9%)	2.8% (2.1–3.7%)	20.7% (18.8–22.7%)
3 years rate	3.6% (2.0–5.9%)	18.4% (14.7–22.5%)	4.0% (3.1–5.0%)	23.3% (21.3–25.4%)
4 years rate	4.0% (2.3–6.4%)	20.2% (16.2–24.5%)	5.3% (4.2–6.5%)	25.8% (23.6–27.9%)
5 years rate	4.4% (2.5–7.0%)	23.4% (19.0–28.1%)	6.7% (5.5–8.1%)	26.5% (24.3–28.7%)
6 years rate	6.0% (3.6–9.2%)	24.9% (20.3–29.8%)	7.3% (5.9–8.8%)	27.6% (25.4–29.9%)
7 years rate	6.7% (4.0–10.2%)	25.6% (20.9–30.6%)	8.2% (6.7–9.9%)	28.1% (25.8–30.5%)
8 years rate	7.8% (4.6–12.0%)	26.7% (21.6–32.0%)	8.7% (7.1–10.5%)	28.6% (26.2–31.0%)
9 years rate	10.4% (5.2–17.6%)	26.7% (21.6–32.0%)	9.0% (7.3–10.9%)	28.6% (26.2–30.1%)

Abbreviations: Confidence interval, CI; major adverse cardiac- and cerebrovascular event, MACCE.

Table S5. Newly detected cancers during follow-up.

**Stratum 1: HD = No**				
**Timelist**	**time_new_cancer**	**Cumulative Incidence**	**95% Confidence Interval**	
**365**	**347**	0,0143	0,00988	0,0201
**730**	**716**	0,0275	0,0210	0,0352
**1095**	**1077**	0,0389	0,0310	0,0482
**1461**	**1434**	0,0502	0,0408	0,0609
**1826**	**1816**	0,0627	0,0518	0,0750
**2190**	**2059**	0,0702	0,0582	0,0835
**2555**	**2544**	0,0793	0,0659	0,0942
**2920**	**2831**	0,0852	0,0705	0,1016
**3285**	**3206**	0,0914	0,0747	0,1102
**3650**	**.**	.	.	.

**Cumulative Incidence Function Estimates**				
**Stratum 2: HD = Yes**				
**Timelist**	**time_new_cancer**	**Cumulative Incidence**	**95% Confidence Interval**	
**365**	**363**	0,1487	0,1338	0,1643
**730**	**728**	0,2003	0,1833	0,2178
**1095**	**1083**	0,2242	0,2063	0,2427
**1461**	**1420**	0,2473	0,2284	0,2666
**1826**	**1777**	0,2593	0,2398	0,2792
**2190**	**2161**	0,2713	0,2510	0,2920
**2555**	**2500**	0,2766	0,2559	0,2977
**2920**	**2846**	0,2823	0,2608	0,3041
**3285**	**2846**	0,2823	0,2608	0,3041
**3650**	**.**	.	.	.

**Gray's Test for Equality of Cumulative Incidence Functions**		
**Chi-Square**	**DF**	**Pr > Chi-Square**
302,2995	1	<.0001

Cox Regressionen/Logistische Regressionen.

Table S6. Drug adherence during the follow-up period based on prescribed medications.

	STEMI—Hematologic Malignancies		NSTEMI—Hematologic Malignancies	
	**No**	**Yes**	**No**	**Yes**
**OAC—% (95% CI)**				
180 days after AMI	12.3% (12.1 – 12.5%)	17.7% (12.8–22.6%)	20.8% (20.6–21.0%)	26.7% (23.9–29.5%)
1 year after AMI	9.7% (9.5 – 9.9%)	12.4% (8.5–16.4%)	17.1% (17.0–17.3%)	23.7% (20.7–26.6%)
2 years after AMI	10.3% (10.1 – 10.5%)	14.9% (9.9–19.9%)	17.9% (17.7–18.1%)	24.3% (20.7–27.9%)
3 years after AMI	11.0% (10.8 – 11.2%)	18.9% (12.3–25.5%)	18.7% (18.5–18.9%)	25.6% (21.6–29.6%)
4 years after AMI	11.8% (11.6–12.0%)	20.8% (13.5–28.2%)	19.3% (19.1–19.6%)	25.7% (20.3–31.0%)
5 years after AMI	12.7% (12.4–13.0%)	21.3% (12.8–29.7%)	20.2% (19.9–20.4%)	27.2% (21.1–33.4%)
**PAI—% (95% CI)**				
180 days after AMI	96.1% (95.8–96.3%)	93.3% (86.3–100.3%)	85.6% (85.4–85.8%)	77.6% (74.3–80.9%)
1 year after AMI	91.6% (91.3–91.8%)	85.8% (77.2–94.3%)	77.1% (76.9–77.3%)	66.6% (62.2–71.0%)
2 years after AMI	65.4% (65.0–65.7%)	62.3% (52.8–71.9%)	56.6% (56.3–56.9%)	48.5% (43.8–53.3%)
3 years after AMI	60.7% (60.3–61.0%)	59.4% (48.6–70.2%)	52.9% (52.6–53.2%)	47.4% (41.8–52.9%)
4 years after AMI	58.4% (57.9–58.8%)	49.7% (38.2–61.1%)	51.2% (50.9–51.5%)	47.2% (40.8–53.6%)
5 years after AMI	56.7% (56.2–57.2%)	51.6% (39.2–64.0%)	49.8% (49.4–50.2%)	43.6% (35.9–51.3%)
**OAC and/or PAI—% (95% CI)**				
180 days after AMI	97.3% (97.0–97.5%)	96.5% (89.3–103.6%)	91.3% (91.1–91.5%)	85.9% (82.0–89.8%)
1 year after AMI	94.0% (93.7–94.3%)	89.3% (81.2–97.4%)	85.3% (85.0–85.5%)	78.7% (73.9–83.5%)
2 years after AMI	72.2% (71.9–72.6%)	72.0% (61.3–82.7%)	69.6% (69.4–69.9%)	67.0% (61.5–72.5%)
3 years after AMI	68.6% (68.3–68.9%)	71.6% (59.8–83.5%)	67.3% (67.0–67.6%)	68.3% (61.0–75.7%)
4 years after AMI	67.1% (66.7–67.5%)	67.2% (54.6–79.8%)	66.3% (66.0–66.6%)	67.9% (60.4–75.5%)
5 years after AMI	66.2% (65.8–66.6%)	68.3% (54.7–81.9%)	65.8% (65.4–66.2%)	65.1% (56.0–74.2%)
**Statins—% (95% CI)**				
180 days after AMI	93.0% (92.7–93.2%)	85.4% (78.1–92.7%)	83.5% (83.3–83.7%)	71.7% (67.9–75.5%)
1 year after AMI	86.1% (85.8–86.4%)	79.6% (71.4–87.7%)	75.6% (75.3–75.8%)	63.2% (58.5–67.8%)
2 years after AMI	83.0% (82.7–83.3%)	73.3% (62.9–83.7%)	74.0% (73.8–74.3%)	61.2% (55.8–66.7%)
3 years after AMI	81.4% (81.1–81.8%)	74.2% (63.1–85.2%)	73.3% (73.0–73.6%)	65.4% (59.0–71.7%)
4 years after AMI	80.6% (80.2–80.9%)	80.0% (67.2–92.8%)	73.2% (72.8–73.5%)	64.1% (56.2–72.0%)
5 years after AMI	80.0% (79.6–80.4%)	79.3% (65.1–93.5%)	73.3% (72.9–73.7%)	63.1% (54.5–71.8%)
**Beta blockers—% (95% CI)**				
180 days after AMI	92.2% (91.9–92.4%)	88.2% (80.5–95.9%)	88.4% (88.1–88.6%)	87.3% (83.5–91.1%)
1 year after AMI	85.2% (84.9–85.5%)	84.4% (75.5–93.4%)	80.8% (80.6–81.1%)	78.7% (74.2–83.2%)
2 years after AMI	82.3% (82.0–82.6%)	81.3% (70.3–92.4%)	79.2% (78.9–79.4%)	77.8% (72.0–83.5%)
3 years after AMI	80.8% (80.5–81.2%)	81.6% (69.5–93.6%)	78.5% (78.2–78.8%)	78.4% (71.2–85.5%)
4 years after AMI	80.1% (79.6–80.5%)	83.3% (70.2–96.4%)	78.3% (77.9–78.6%)	78.2% (69.8–86.5%)
5 years after AMI	79.8% (79.4–80.2%)	84.9% (71.0–98.8%)	77.9% (77.5–78.3%)	76.7% (67.1–86.4%)
**ACE-inhibitors/ ARB—% (95% CI)**				
180 days after AMI	92.3% (92.0–92.5%)	90.9% (82.8–99.1%)	90.2% (90.0–90.4%)	85.0% (81.5–88.5%)
1 year after AMI	82.7% (82.4–82.9%)	75.5% (66.5–84.6%)	79.7% (79.5–80.0%)	68.2% (64.1–72.3%)
2 years after AMI	80.4% (80.1–80.7%)	72.6% (61.8–83.4%)	78.1% (77.8–78.3%)	66.5% (61.3–71.6%)
3 years after AMI	79.1% (78.8–79.5%)	75.3% (62.6–87.9%)	77.3% (77.0–77.6%)	64.3% (58.0–70.5%)
4 years after AMI	78.5% (78.1–78.9%)	68.1% (55.1–81.0%)	76.9% (76.5–77.2%)	63.7% (56.0–71.3%)
5 years after AMI	78.2% (77.7–78.6%)	75.4% (61.4–89.4%)	76.4% (76.1–76.8%)	62.6% (53.3–71.9%)

Abbreviations: Angiotensin-converting enzyme, ACE; acute myocardial infarction, AMI; angiotensin receptor blocker, ARB; coronary artery bypass grafting, oral anticoagulants, OAC; platelet activation inhibition, PAI.

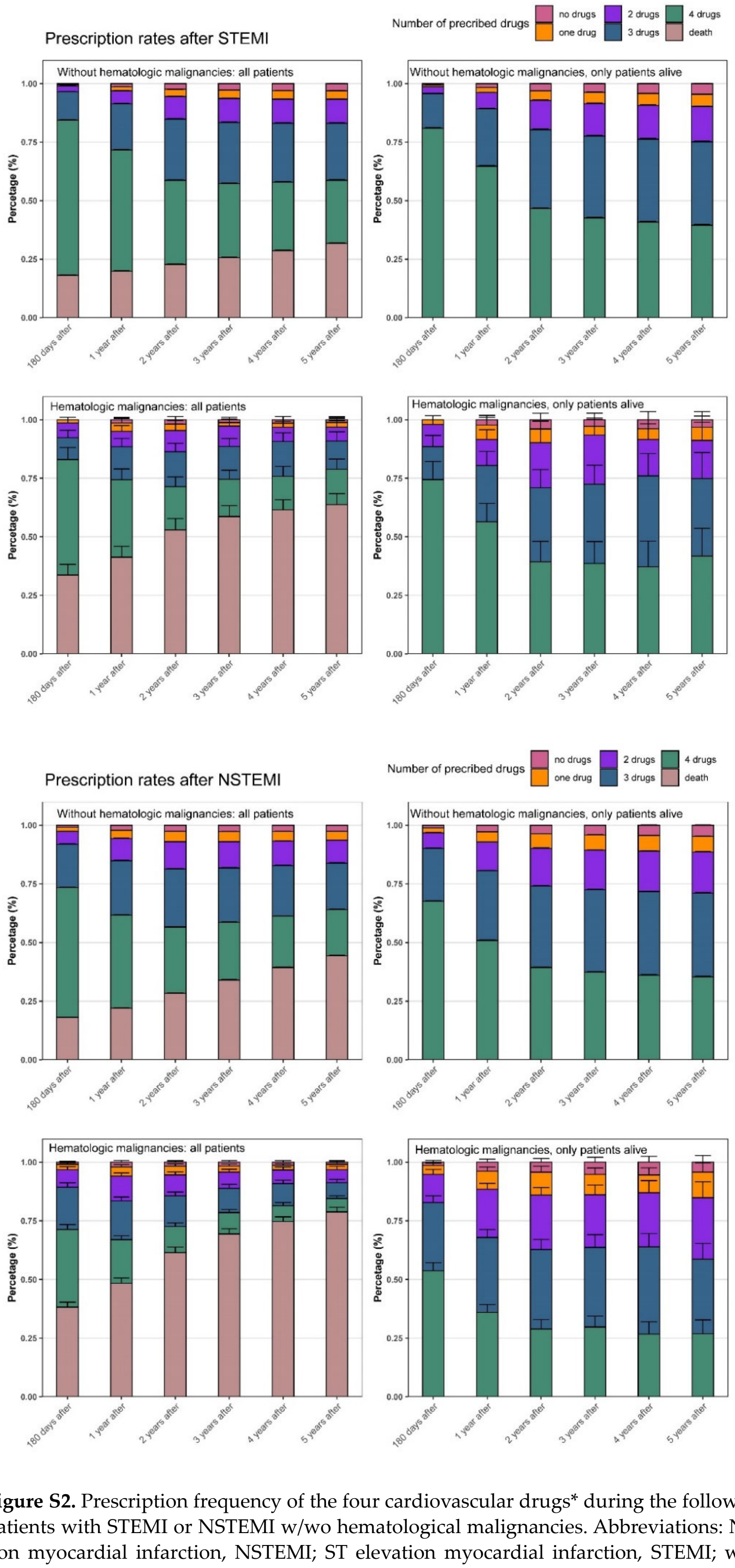

Figure S2. Prescription frequency of the four cardiovascular drugs* during the follow-up period in patients with STEMI or NSTEMI w/wo hematological malignancies. Abbreviations: Non-ST elevation myocardial infarction, NSTEMI; ST elevation myocardial infarction, STEMI; w/wo, with or

without. * Angiotensin converting enzyme blocker or angiotensin receptor blocker, oral anticoagulants or platelet activation inhibition, statin and beta blocker.

Table S7. Incidence for various hematologic malignancies divided into 8 subgroups in Germany from 2011 to 2019. Data received by the Federal Statistical Offices [DESTATIS]; https://www.destatis.de, 23 March 2023)**.**

	Incidence								
**Year**	Group 1	Group 2	Group 3	Group 4	Group 5	Group 6	Group 7	Group 8	all_HM
2011	4.3	5.5	7.55	20.47	18.23	1.84	4.09	18.07	80.7
2012	4.12	6.18	7.92	21.13	18.61	2.03	4.32	18.78	83.71
2013	4.26	6.38	8.68	21.93	18.92	1.9	4.54	19.36	86.65
2014	4.16	6.96	9.02	21.88	19.19	2	4.99	20.27	89.09
2015	4.13	6.66	9.24	22.43	19.38	2.1	5.14	20.16	89.99
2016	4.33	6.93	9.81	23.11	19.55	2.06	5.34	20.43	92.31
2017	4.01	7.11	9.48	21.88	19.44	1.79	5.45	19.48	89.38
2018	3.83	7.16	9.32	21.51	19.42	1.52	5.31	19.65	88.36
2019	3.72	7.09	9.23	21.41	19.64	1.41	5.44	19.47	88.08
**mean**	4.10	6.70	8.97	21.75	19.15	1.85	4.96	19.52	87.59
**SD**	0.21	0.55	0.74	0.76	0.47	0.24	0.51	0.75	3.49

Abbreviations: hematology malignancies, hm; standard deviation, SD. Group 1: Hodgkin-Lymphoma [Lymphogranulomatosis], C81.-; Group 2: Follicular lymphoma, Non- Follicular lymphoma, C82.-; C83.0; Group 3: Aggressive lymphoma, C83.3; C83.5; C83.7; C83.8; Group 4: Plasmocytoma and malignant plasma cell neoplasm, C90.x0; C90.x1; Group 5: Lymphocytic leukemia, C91.10; C91.11; Group 6: Myeloid leukemia, monocytic leukemia, other leukemias of specified cell type, C92.3x; C92.5x; C92.6x; C92.7x; C92.8x; C92.9x; C93.0x; C93.7x; C94.0x; C94.2x; Group 7: Chronic myeloid leukemia, C92.1x; and Group 8: Myelodysplastic and myeloproliferative diseases, unclassifiable, Myelodysplastic syndromes.

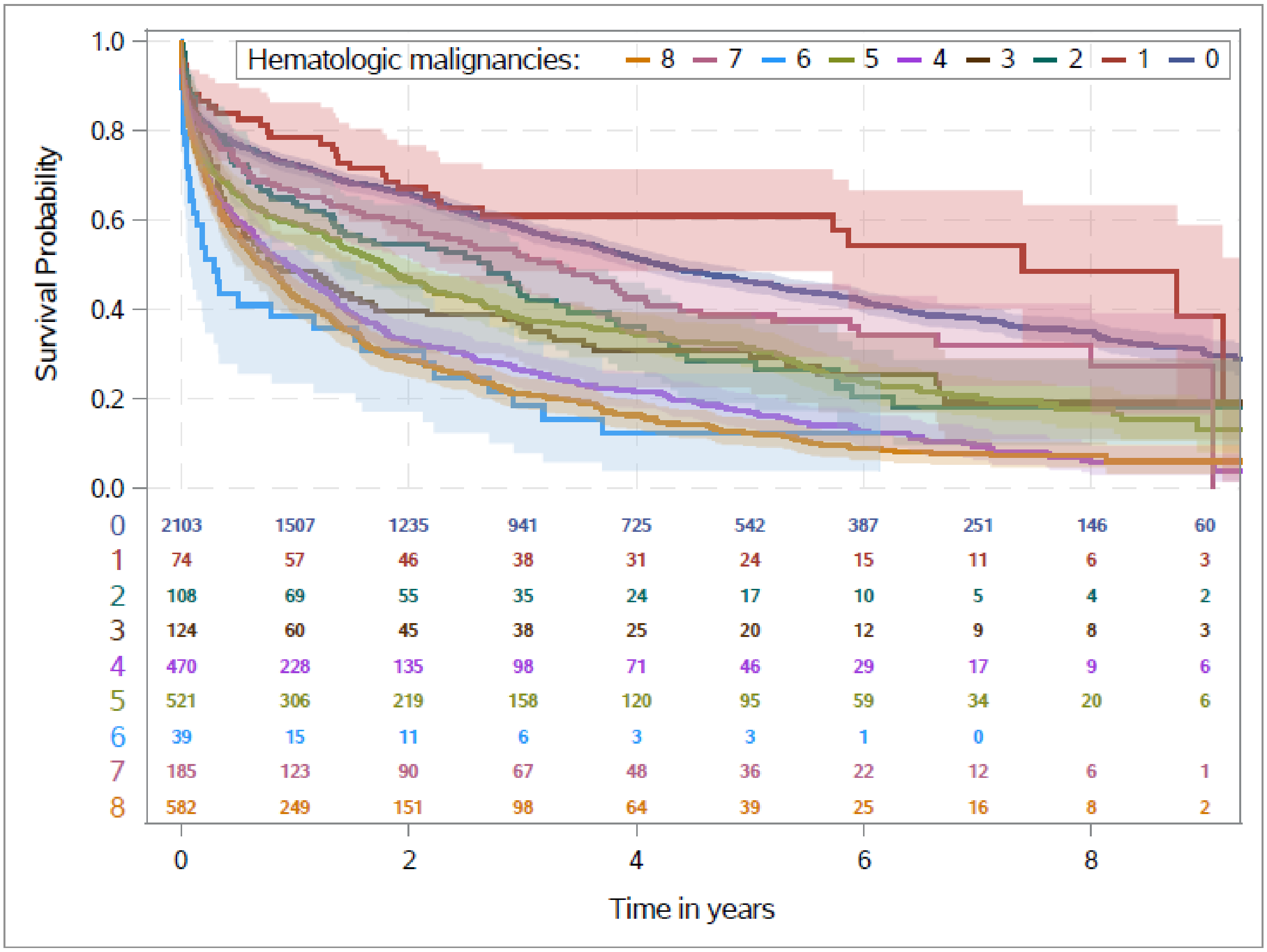

Figure S3. Caplan- Meier Survival in ACS depending on hematology malignancies.

0. and no Cancer (no C* code in history);
1. Hodgkin's disease C81.-;
2. Follicular lymphoma, Non-Follicular lymphoma, C82.-; C83.0;
3. Aggressive lymphoma, C83.3; C83.5; C83.7; C83.8;
4. Plasmocytoma and malignant plasma cell neoplasm, C90.x0; C90.x1;
5. Lymphocytic leukemia {group 5}, C91.10; C91.11;
6. Myeloid leukemia, monocytic leukemia, other leukemias of specified cell type, C92.3x; C92.5x; C92.6x; C92.7x; C92.8x; C92.9x; C93.0x; C93.7x; C94.0x; C94.2x;
7. Chronic myeloid leukemia, C92.1x;
8. Myelodysplastic and myeloproliferative diseases, unclassifiable, Myelodysplastic syndromes; C94.6x; D46.-.

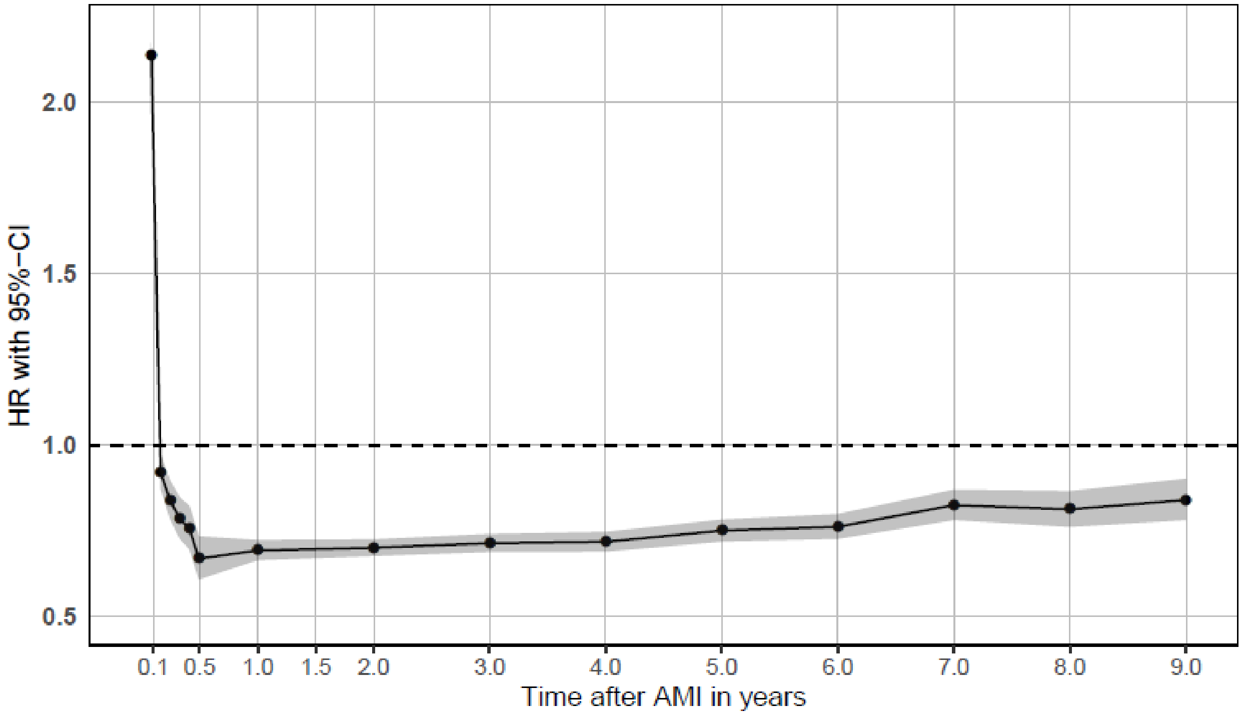

Time	1 month	6 months	1 year	3 years	5 years	7 years	≥9 years
HR	2.14	0.92	0.69	0.71	0.75	0.83	0.84
95% CI	2.10 – 2.17	0.87 – 0.97	0.67 – 0.723	0.69 – 0.75	0.72 – 0.69	0.78 – 0.87	0.78 – 0.90
P value	<0.001	<0.001	<0.001	<0.001	<0.001	<0.001	<0.001

Figure S4. Survival in STEMI vs. NSTEMI. In the early phase, there is a survival disadvantage in patients with STEMI, which rapidly shifts to the disadvantage of NSTEMI patients in the first months and then permanently. Abbreviations: Acute myocardial infarction, AMI; Confidence interval, CI; hazard ratio, HR; non ST-elevation myocardial infarction, NSTEMI; ST-elevation myocardial infarction, STEMI.
